# Exploring the causal relationship between body mass index and keratoconus: a Mendelian randomization study

**DOI:** 10.3389/fmed.2024.1402108

**Published:** 2024-07-10

**Authors:** Jiaoman Wang, Fangyuan Liu, Jianhao Mo, Di Gong, Fang Zheng, Jingjing Su, Sicheng Ding, Weihua Yang, Ping Guo

**Affiliations:** ^1^The 2nd Clinical Medical College of Jinan University, Shenzhen, China; ^2^Shenzhen Eye Institute, Shenzhen Eye Hospital, Jinan University, Shenzhen, China; ^3^Department of Ophthalmology, Jinzhou Medical University, Jinzhou, China

**Keywords:** keratoconus, BMI, obesity, Mendelian randomization, inflammatory factors

## Abstract

**Background:**

Despite reports suggesting a link between obesity and keratoconus, the causal relationship is not fully understood.

**Methods:**

We used genome-wide association study (GWAS) data from public databases for a two-sample Mendelian randomization analysis to investigate the causal link between body mass index (BMI) and keratoconus. The primary method was inverse variance weighted (IVW), complemented by different analytical techniques and sensitivity analyses to ensure result robustness. A meta-analysis was also performed to bolster the findings’ reliability.

**Results:**

Our study identified a significant causal relationship between BMI and keratoconus. Out of 20 Mendelian randomization (MR) analyses conducted, 9 showed heterogeneity or pleiotropy. Among the 11 analyses that met all three MR assumptions, 4 demonstrated a significant causal difference between BMI and keratoconus, while the remaining 7 showed a positive trend but were not statistically significant. Meta-analysis confirmed a significant causal relationship between BMI and keratoconus.

**Conclusion:**

There is a significant causal relationship between BMI and keratoconus, suggesting that obesity may be a risk factor for keratoconus.

## 1 Introduction

Keratoconus (KC) is a relatively common congenital developmental anomaly characterized by thinning of the central or paracentral cornea, increased curvature, bilateral asymmetry, and progressive expansion. It initially presents as myopia and irregular astigmatism, leading to vision loss in later stages due to corneal stromal scarring, protrusion, and thinning (1, 2). Globally, its prevalence is estimated to be between 1:375 and 1:2,000 individuals, with a significantly higher rate among younger populations (3). In the early stages, the progression of keratoconus can be slowed with collagen cross-linking, while later stages may require corneal transplantation to restore vision (3). Therefore, identifying other modifiable risk factors for keratoconus is crucial for its prevention and management.

However, the pathogenesis of keratoconus is still unclear, but it is generally considered a multifactorial disease influenced by environmental, genetic, metabolic, viral infection, lifestyle, and immune factors (4, 5).

Body mass index (BMI, kg/m^2^) is commonly used to measure obesity, with BMI ≥25 considered overweight and BMI ≥30 classified as obese (6). Previous studies have shown that an increase in BMI is associated with a variety of diseases. At a systemic level, higher BMI is linked to a greater risk of type 2 diabetes, cardiovascular diseases, and sleep apnea (7, 8). Locally, when focusing on the eyes, higher BMI is also significantly associated with age-related cataracts, age-related macular degeneration, and diabetic retinopathy (9, 10).

To date, the impact of high BMI on keratoconus remains unclear. Past reports have indicated that in Australian patients (11), a higher BMI was not associated with an increased risk of keratoconus; a recent study in Iran (12) found no significant difference in BMI between keratoconus patients and control subjects. However, recent findings suggest that a high BMI may be a risk factor for keratoconus. Research by Eliasi et al. (13) indicated that in the adolescent population, those who are overweight (OR = 1.42, *p* < 0.05) and obese (OR = 1.50, *p* < 0.05) have a higher likelihood of developing keratoconus; Ren et al. (14) suggested that individuals with a high BMI may be more prone to eye rubbing, thereby increasing the risk of KC. Clarifying the causal relationship between high BMI and keratoconus could enhance strategies for keratoconus detection and prevention. However, despite reports of a significant correlation between obesity and keratoconus, the causal link remains unclear due to the influence of confounding factors and the limited number of related studies.

Mendelian randomization (MR) is an emerging method (15) for testing causal relationships between risk factors and various phenotypes. The fundamental principle involves inferring the influence of biological factors on disease by utilizing the effects of genotypes, which are randomly distributed in nature, on phenotypes. Using genetic variants as instrumental variables can effectively reduce biases and confounding factors, thereby increasing the accuracy of causal inference.

To ascertain whether there is a causal relationship between obesity and keratoconus, this study intends to use the two-sample Mendelian randomization (MR) analysis approach to explore the causal link between BMI and keratoconus.

## 2 Workflow and study design

**Figure 1:**
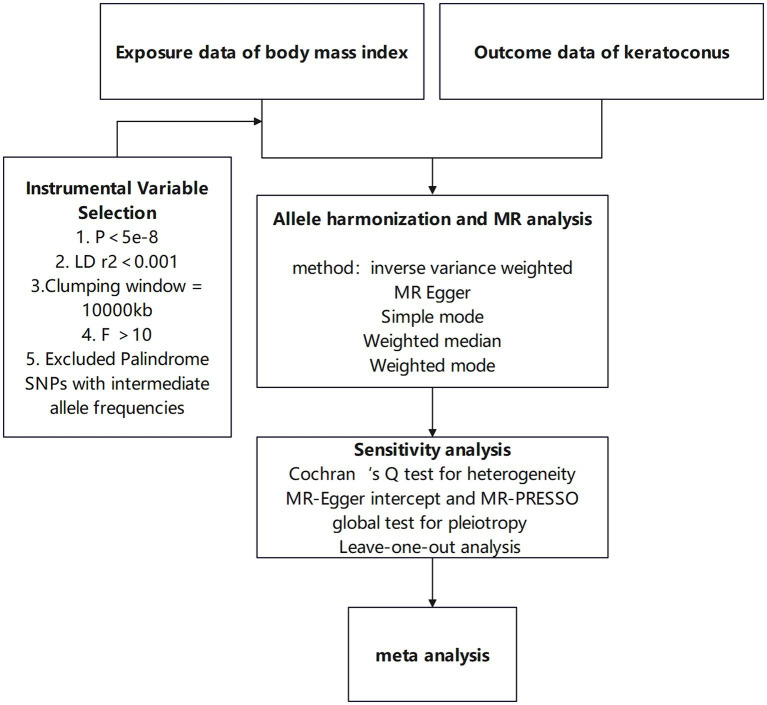
Workflow of the study.

**Figure 2:**
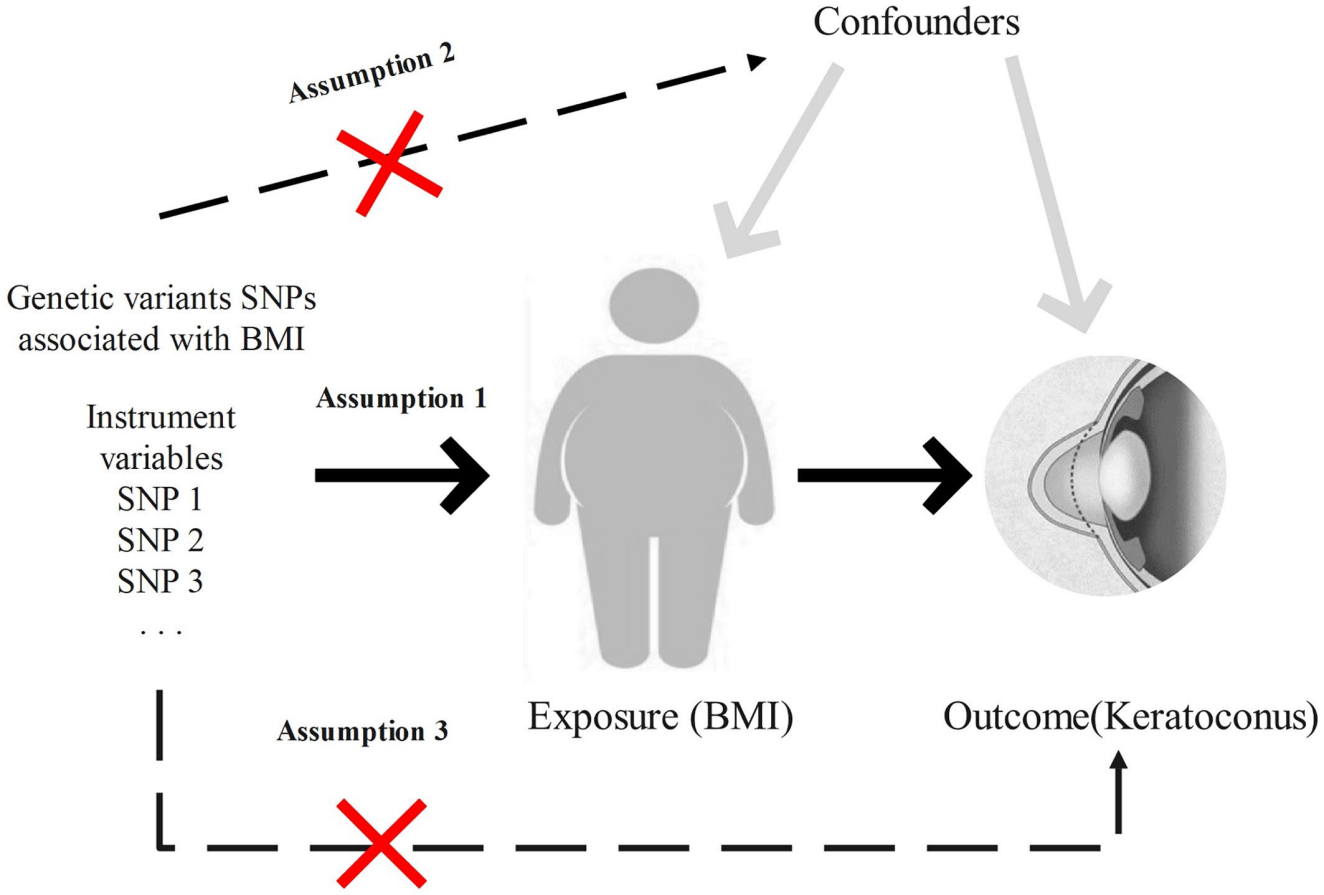
Study design and key assumptions of Mendelian randomization. Assumption 1: the instrumental variables are closely related to the exposure. Assumption 2: the instrumental variables are not associated with any confounding factors. Assumption 3: the instrumental variables, aside from exposure, are not directly related to keratoconus.

This study utilizes a two-sample Mendelian randomization approach to investigate the causal relationship between BMI and keratoconus, enhancing result robustness through heterogeneity tests and horizontal pleiotropy tests. Meta-analysis is subsequently used to further ensure the robustness of the findings. The specific workflow is shown in Figure 1. This research employs various BMI-associated single-nucleotide polymorphisms (SNPs) as instrumental variables to assess the causal relationship between BMI and keratoconus. MR analysis must satisfy the following three assumptions: first, the instrumental variables related to the exposure must be closely related to the exposure itself; second, the selected instrumental variables must not be related to any confounding factors between the exposure and outcome; and third, the instrumental variables can only influence the outcome through the exposure, meaning in this study, the chosen instrumental variables can only affect keratoconus through BMI. The study design is illustrated in Figure 2.

## 3 Materials and methods

### 3.1 Data sources

GWAS data related to keratoconus were sourced from the GWAS Catalog^1^ and Finngen R9.^2^ The dataset from the GWAS Catalog was obtained from a large-scale GWAS, for which we used the first-phase meta-analysis, including 2,116 cases and 24,626 European ancestry controls. The GWAS data from Finngen R9 included 311 cases and 209,287 European ancestry controls.

To acquire as much BMI-related GWAS data as possible, this study searched Open GWAS^3^ using “body mass index” as the search term, obtaining all BMI-related GWAS data for European populations. After eliminating duplicate studies, 10 BMI-related GWAS datasets were used for further research, with GWAS IDs being ebi-a-GCST006368, ebi-a-GCST90018947, ebi-a-GCST90025994, ebi-a-GCST90029007, ieu-a-85, ieu-a-835, ieu-a-1089, ieu-b-40, ukb-a-248, and ukb-b-2303. Detailed information about all the BMI-related GWAS datasets is presented in Table 1.

**Table 1:** Detailed information on GWAS data related to BMI.

ID	PMID	Population	Sample size
ieu-b-40	30124842 (16)	European	681,275
ebi-a-GCST90029007	29892013 (17)	European	532,396
ebi-a-GCST90025994	34226706 (18)	European	457,756
ukb-b-2303		European	454,884
ebi-a-GCST90018947	34594039 (19)	European	359,983
ukb-a-248		European	336,107
ieu-a-835	25673413 (20)	European	322,154
ebi-a-GCST006368	30108127 (21)	European	315,347
ieu-a-1089	26961502 (22)	European	120,286
ieu-a-85	23563607 (23)	European	16,068

### 3.2 Instrumental variable selection

To select instrumental variables highly related to BMI, we filtered SNPs based on a standard of *p* < 5 × 10^−8^, relaxing the criterion to *p* < 5 × 10^−6^ if fewer than 5 SNPs were selected. To ensure the independence between SNPs. We removed linkage disequilibrium based on an *R*^2^ < 0.001 and distance of Kb < 10,000. Furthermore, weak instrumental variables were eliminated if they had an *F* value (*F* = (β_exposure_/SE_exposure_)^2^, where β_exposure_ and SE_exposure_ are the effect value and standard error of the exposure, respectively) <10. Finally, all palindromic SNPs with intermediate allele frequencies were excluded.

### 3.3 Mendelian randomization analysis

This study employed a two-sample Mendelian randomization approach to explore the causal relationship between BMI and keratoconus, with weighted median as the primary analysis method, supplemented by MR Egger, simple mode, weighted median, and weighted mode as auxiliary analysis methods. Cochran’s *Q* test was used to assess the heterogeneity of the instrumental variables, with *p* > 0.05 indicating no heterogeneity; MR PRESSO and MR Egger’s intercept tests were used to determine the presence of horizontal pleiotropy, with *p* > 0.05 suggesting no pleiotropy, among them, MRPRESSO can detect the residual validity and outliers of MR analysis, which can correct horizontal pleiotropy by removing outliers and further evaluate the robustness of causal relationships. Additionally, leave-one-out sensitivity analysis was conducted to determine if the results were driven by a single strong SNP.

### 3.4 Meta-analysis

This study incorporated results that met the three fundamental assumptions of MR analysis into meta-analysis to ensure the robustness of the findings. Before conducting meta-analysis, heterogeneity testing was performed on all results. If significant heterogeneity was observed among the results (*I*^2^ > 50%), a random-effects model was used; otherwise, a fixed-effects model was employed.

### 3.5 Data analysis

All analyses were conducted using R 4.3.1 and relevant R packages including “TwoSampleMR (0.5.7),” “MRPRESSO (1.0),” and “metafor.”

## 4 Result

### 4.1 Obtaining instrumental variables

After stringent selection and removal of linkage disequilibrium, this study acquired 10 sets of SNPs significantly associated with BMI, with the number of SNPs per set ranging from 7 to 475. Detailed data on the instrumental variables used for MR analysis in each dataset can be found in Supplementary material S1.

### 4.2 Mendelian randomization analysis

This study conducted a total of 20 MR analyses (10 for BMI X 2 for KC), and after excluding 9 results with heterogeneity and horizontal pleiotropy, 11 were considered to have neither. Detailed results of Cochran’s *Q* test, MR PRESSO, and MR Egger’s intercept test can be found in Supplementary material S2. Interestingly, among all results, 4 indicated a significant causal relationship between BMI and KC, with outcome data for these 4 results all coming from the FinnGen R9 database.

**Figure 3:**
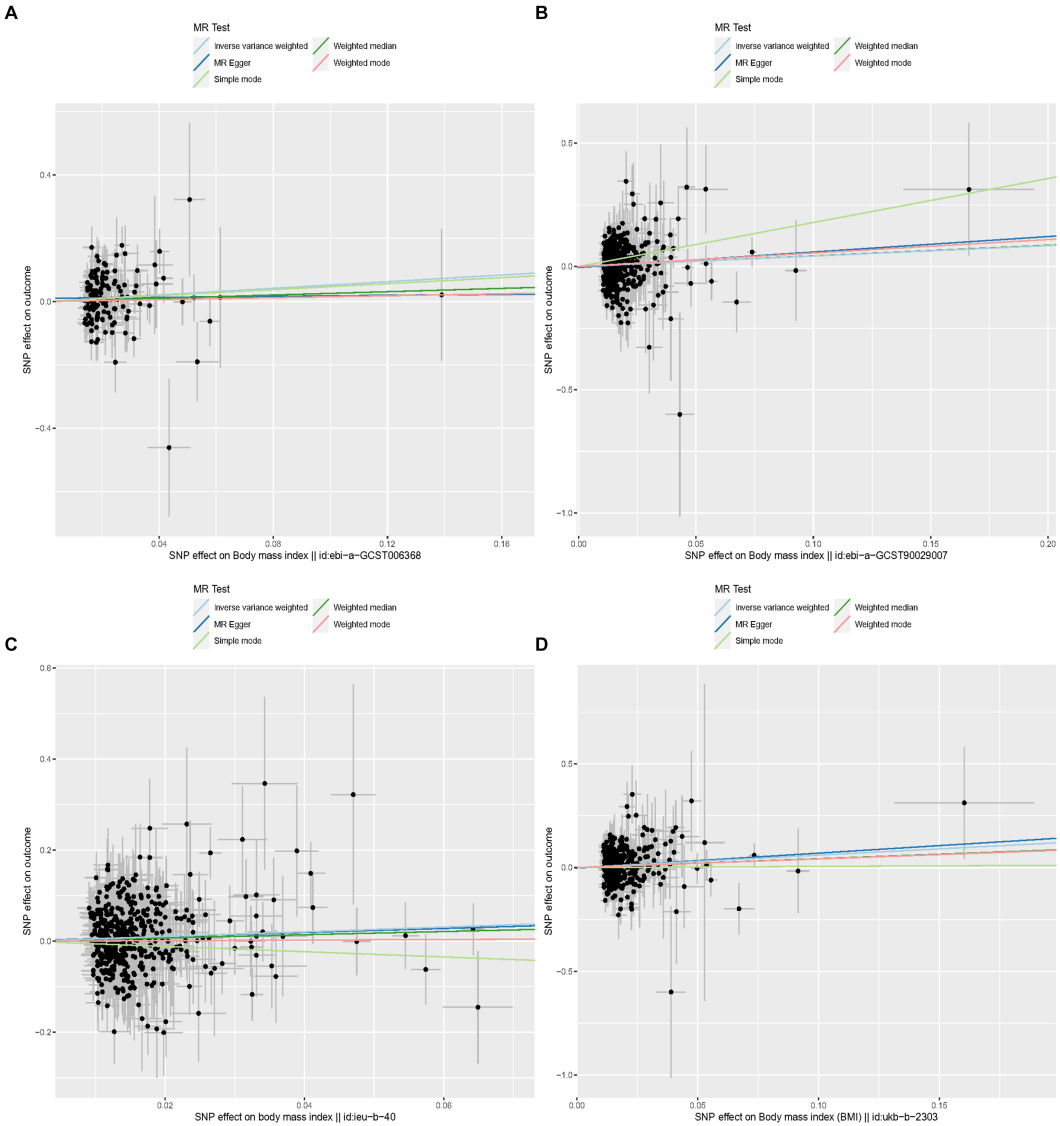
Scatter plots for MR analyses of the causal effect of proinsulin levels on keratoconus. Each line shows the slope corresponding to the estimated MR effect each method. **(A)** The ID number of the GWAS data related to BMI is ebi-a-GCST006368. **(B)** The ID number of the GWAS data related to BMI is ebi-a-GCST90029007. **(C)** The ID number of the GWAS data related to BMI is ieu-b-40. **(D)** The ID number of the GWAS data related to BMI is ukb-b-2303.

**Figure 4:**
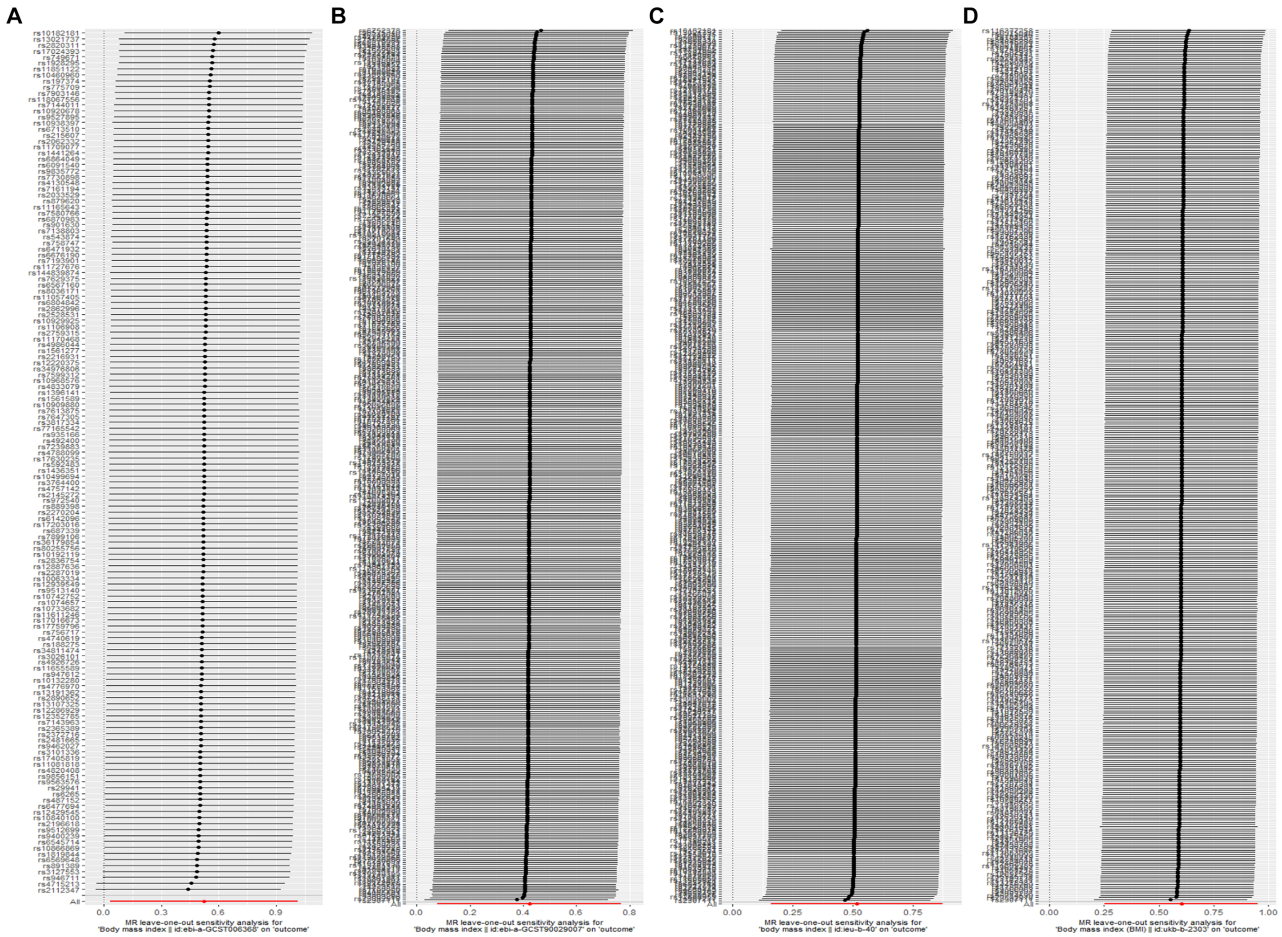
Leave-one-out sensitivity analysis shows that the results are not caused by a strong single SNP. **(A)** The ID number of the GWAS data related to BMI is ebi-a-GCST006368. **(B)** The ID number of the GWAS data related to BMI is ebi-a-GCST90029007. **(C)** The ID number of the GWAS data related to BMI is ieu-b-40. **(D)** The ID number of the GWAS data related to BMI is ukb-b-2303.

Specifically, when the exposure data came from a GWAS by Hoffmann et al. (21) involving 315,347 individuals of European ancestry, the OR (IVW) was 1.69, 95% CI was 1.03 to 2.76, *p* = 0.037. Although not statistically significant, the OR values for MR Egger, simple mode, weighted mode, and weighted median were all >1, consistent with the IVW method. When the exposure data was from Loh et al. (17) involving 532,396 individuals of European ancestry, the OR (IVW) was 1.53, 95% CI was 1.08 to 2.16, *p* = 0.016, with OR values for the other four auxiliary methods also >1. In the MR analysis of data from Yengo et al. (16) involving 681,275 individuals, the OR (IVW) was 1.68, 95% CI was 1.17 to 2.39, *p* = 0.005, even though the other four methods did not show statistically significant results. In the MR analysis of data from Ben Elsworth et al. involving 454,884 individuals of European ancestry, the OR (IVW) was 1.83, 95% CI was 1.24 to 2.59, *p* = 0.001, with the other four methods not showing statistically significant results, but again, all OR values were >1, consistent with the IVW method trend. Scatter plots for all results are shown in Figure 3. Leave-one-out sensitivity analysis results are presented in Figure 4.

### 4.3 Meta analysis

**Figure 5:**
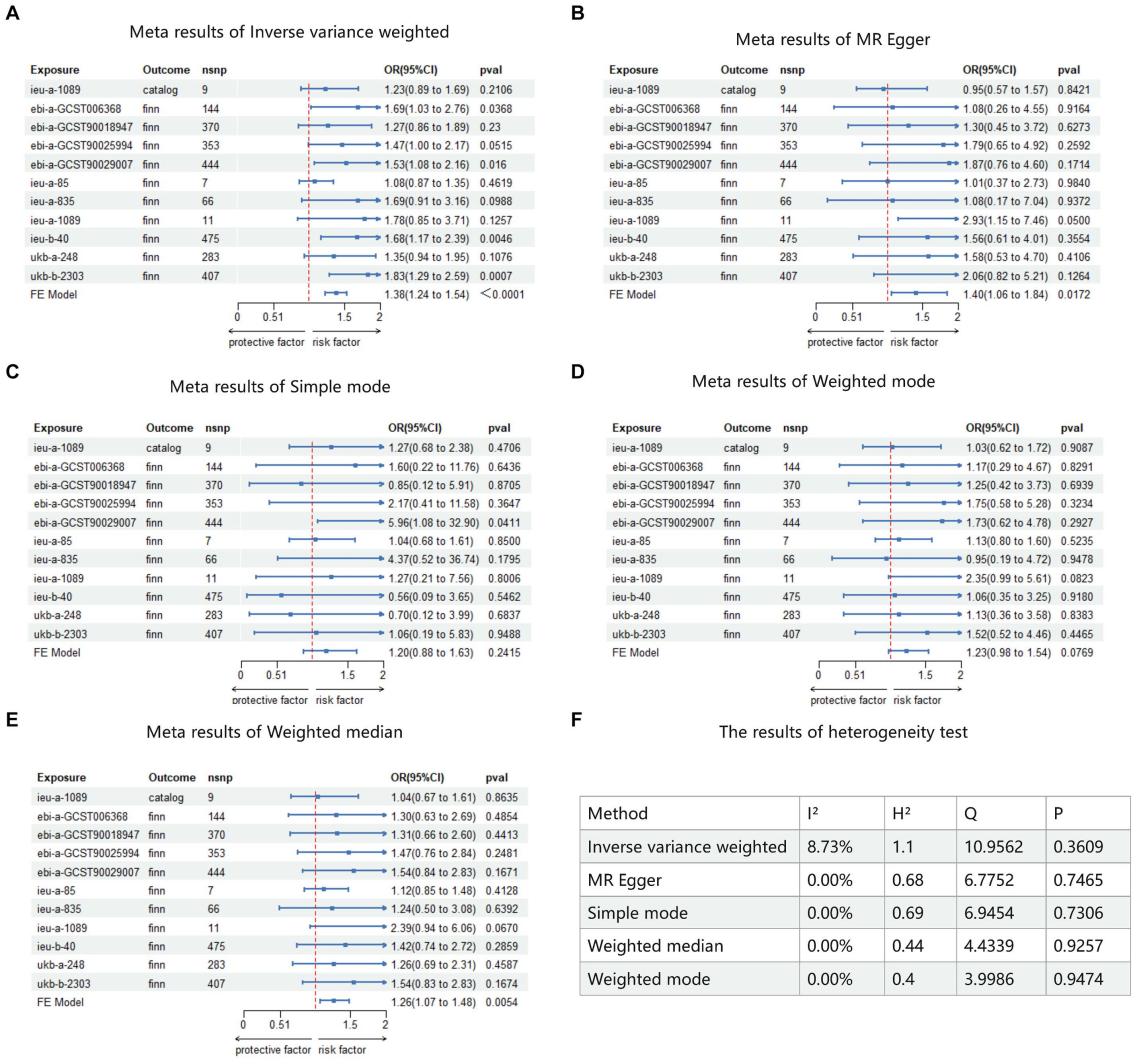
Meta analysis result of MR. Catalog: GWAS data for KC sourced from GWAS Catalog. Finn: GWAS data for KC sourced from FinnGen R9. FE model: fixed-effects model used for meta-analysis. **(A)** Meta analysis result of inverse variance weighted. **(B)** Meta analysis result of MR Egger. **(C)** Meta analysis result of simple mode. **(D)** Meta analysis result of weighted mode. **(E)** Meta analysis result of weighted median. **(F)** The result of heterogeneity test.

This study included these 11 results in a meta-analysis. Heterogeneity testing revealed no heterogeneity among all results, thus a fixed-effects model was adopted for the meta-analysis. The results showed that BMI is a risk factor for keratoconus. IVW: OR = 1.38, 95% CI (1.238–1.54), *p* < 0.0001 (Figure 5A); MR Egger: OR = 1.40, 95% CI (1.06–1.84), *p* = 0.0172 (Figure 5B); simple mode: OR = 1.20, 95% CI (0.88–1.63), *p* = 0.2415 (Figure 5C); weighted mode: OR = 1.23, 95% CI (0.98–1.54), *p* = 0.076 (Figure 5D); weighted median: OR = 1.26, 95% CI (1.07–1.48), *p* = 0.0054 (Figure 5E). Detailed results of heterogeneity testing for the meta-analysis are shown in Figure 5F. Furthermore, the funnel plots can be found in Supplementary Figures S1–S4.

## 5 Discussion

Given the low natural incidence rate and lengthy disease course of keratoconus (KC), it is challenging to conduct prospective studies on the causal relationship between keratoconus and BMI in the real world. Retrospective studies are susceptible to confounding factors and reverse causality, which can render the results unreliable. Therefore, this study employs genetic variants related to BMI as instrumental variables, utilizing “natural random allocation” to assess whether these genes are associated with the risk of keratoconus. This approach helps to eliminate the influence of confounding factors and reverse causality that may exist in observational studies, providing more reliable evidence of causality.

In this study, the results of 11 Mendelian randomization (MR) analyses appeared inconsistent, possibly due to differences in sequencing results and study populations. However, the meta-analysis ultimately confirmed BMI as a risk factor for KC, with an ORIVW of 1.38, 95% CI (1.24–1.54), *p* < 0.001. This means for each unit increase in BMI, the risk of developing keratoconus increases by 38%. This suggests a strong causal relationship between higher BMI values and keratoconus, implying that individuals with obesity are at a higher risk of developing KC.

Although the pathophysiological mechanisms by which obesity causes keratoconus are not yet clear, past research has proposed several potential mechanisms.

Inflammatory responses may be a significant cause of keratoconus induced by obesity. Obesity is a systemic chronic metabolic inflammation (24), with upregulation of inflammatory markers such as tumor necrosis factor (TNF-α), interleukins (IL)-6, IL-1β, and CCL2 in the adipose tissue of obese individuals (24, 25). Additionally, organs such as the liver, pancreas, brain, and muscles experience increased exposure to inflammation in the state of obesity, leading to the development of various diseases (26). This chronic inflammation persists over time, and when such “inflammation” occurs in the eye, it can lead to changes in ocular inflammation, resulting in chronic damage to the ocular surface (25). Traditionally, keratoconus (KC) has been viewed as a non-inflammatory disease (27). However, with more in-depth research in recent years, a close association between the onset of KC and inflammation has been discovered (28). Previous studies have confirmed an increased expression of various inflammatory markers such as interleukins, metalloproteinases, and tumor necrosis factors in the corneal tissues and tear samples of patients with keratoconus (29–31). Interleukin 1β (IL-1β) and tumor necrosis factor α (TNF-α) have been shown to reduce the synthesis of collagen, induce apoptosis in corneal cells, and increase the activity of matrix metalloproteinases (MMPs) (31). Interestingly, the expression of MMPs in obese individuals is significantly higher compared to that in normal individuals. Mazor et al. (32) observed an increased expression and activity of MMP-2 in the brain tissues of obese patients, which in turn can reflexively promote obesity. MMPs, including MMP-1, MMP-3, MMP-7, and MMP-13, are enzymes secreted in response to cytokines and growth factors, responsible for the degradation of extracellular matrix proteins (31). In patients with keratoconus (KC), an increase in the proteolytic activity and expression levels of various matrix metalloproteinases (MMPs) and cytokines has been observed compared to healthy controls (29). Compared to healthy individuals and those with other corneal diseases, MMP levels are elevated, and levels of protease inhibitors are reduced in keratoconus specimens (33, 34), which may be a significant factor in the development of KC due to obesity. Furthermore, Dou et al. (35) have discovered through single-cell sequencing that inflammatory markers such as interleukins, metalloproteinases, and chemokines (e.g., CXCL1) are elevated in keratoconus tissue, further confirming the association between the onset of keratoconus and chronic inflammatory responses. Thus, chronic inflammation might be the underlying connection between obesity and the development of keratoconus.

Increased frequency of eye rubbing may be a key factor in the development of keratoconus due to obesity. Previous research indicates that eye rubbing can alter the mechanical stress on the cornea, leading to increased friction between the cornea and the conjunctiva, which causes remodeling of the corneal stromal microstructure and reduces its mechanical stability (36). Research by Dou et al. (35) found that the expression of biomechanical homeostasis regulators, including YAP1 and TEAD1, is elevated in the corneal group of patients with keratoconus, suggesting a biomechanical homeostasis imbalance in the corneal tissues of these patients. Moreover, an increased frequency of eye rubbing can promote the expression of various protease genes in the corneal matrix, including MMP1, MMP3, CTSD, CTSK, etc. (35, 37). This indicates that eye rubbing can not only change the mechanical stress on the cornea but also trigger biochemical reactions, causing an imbalance in corneal matrix protein dissolution and inducing the occurrence of keratoconus.

Recent reports have indicated that high BMI values can lead to damage to corneal epithelial nerves and an increase in dendritic cells in the cornea (38), suggesting that obesity can not only directly cause changes in corneal nerve structures and inflammatory states but also lead to eye discomfort, increase the frequency of eye rubbing in patients, and raise the risk of developing keratoconus. Additionally, it has been reported that obese patients are more prone to eyelid laxity and dermatochalasis compared to healthy individuals, which increases mechanical stress on the cornea from the eyelids, thereby increasing the risk of keratoconus development (13).

Despite recent studies focusing on the relationship between obesity and keratoconus, their findings have not fully ruled out other confounding factors and selection biases. Mendelian randomization (MR) analysis, however, akin to randomized controlled trials, is less susceptible to biases arising from confounding, selection biases, and reverse causality, thereby minimizing the limitations of previous research. Nevertheless, this study still has certain limitations. Firstly, although genetic predictions suggest that high BMI may be one of the risk factors for keratoconus, this study did not provide a detailed explanation of the pathological and physiological mechanisms involved. Secondly, as the GWAS sequencing data for keratoconus predominantly stem from individuals of European ancestry, the MR analysis in this study is limited to European populations, hence the results may not be fully generalizable to other ethnicities.

This study identified a significant causal relationship between high BMI, indicative of obesity, and keratoconus in the European population using Mendelian randomization (MR) methodology, and demonstrated the robustness of this association through various sensitivity analysis methods. Further meta-analysis validated the study findings, further strengthening the causal relationship between obesity and keratoconus. The findings of this study suggest that obesity may be a risk factor for keratoconus in the European population. In clinical management activities targeting obese patients, greater attention should be paid to the potential impact of obesity on ocular health. Furthermore, future research efforts are anticipated to involve more comprehensive whole-genome sequencing data and large-scale multicenter studies to validate the biological mechanisms underlying the association between obesity and keratoconus, elucidating the pathogenesis of keratoconus in greater detail for improved prevention and treatment of both conditions.

## Data availability statement

The original contributions presented in the study are included in the article/Supplementary material, further inquiries can be directed to the corresponding authors.

## Ethics statement

Ethical approval was not required for the studies involving humans because all data obtained from publicly available databases. The studies were conducted in accordance with the local legislation and institutional requirements. Written informed consent for participation was not required from the participants or the participants’ legal guardians/next of kin in accordance with the national legislation and institutional requirements because all data obtained from publicly available databases.

## Author contributions

JW: Conceptualization, Methodology, Writing – original draft, Writing – review & editing. FL: Conceptualization, Data curation, Writing – original draft, Writing – review & editing. JM: Formal analysis, Writing – original draft. DG: Software, Writing – original draft. FZ: Data curation, Writing – original draft. JS: Data curation, Visualization, Writing – review & editing. SD: Data curation, Writing – original draft. WY: Supervision, Writing – review & editing. PG: Supervision, Writing – review & editing.

## Footnotes

^1^ https://www.ebi.ac.uk/gwas/

^2^ https://www.finngen.fi/

^3^ https://gwas.mrcieu.ac.uk/

## References

[1] Meyer JJ Gokul A Vellara HR McGhee CNJ. Progression of keratoconus in children and adolescents. Br J Ophthalmol. (2023) 107:176–80. doi: 10.1136/bjophthalmol-2020-316481, PMID: 34479856

[2] Jones-Jordan LA Walline JJ Sinnott LT Kymes SM Zadnik K. Asymmetry in keratoconus and vision-related quality of life. Cornea. (2013) 32:267–72. doi: 10.1097/ICO.0b013e31825697c4, PMID: 22825402 PMC3482277

[3] Deshmukh R Ong ZZ Rampat R Alió del Barrio JL Barua A Ang M. Management of keratoconus: an updated review. Front Med. (2023) 10:1212314. doi: 10.3389/fmed.2023.1212314, PMID: 37409272 PMC10318194

[4] Song M Fang QY Seth I Baird PN Daniell MD Sahebjada S. Non-genetic risk factors for keratoconus. Clin Exp Optom. (2023) 106:362–72. doi: 10.1080/08164622.2022.2062222, PMID: 35504720

[5] Chen S Li XY Jin JJ Shen RJ Mao JY Cheng FF. Genetic screening revealed latent keratoconus in asymptomatic individuals. Front Cell Dev Biol. (2021) 9:650344. doi: 10.3389/fcell.2021.650344 34136477 PMC8202288

[6] Garrow JS. Three limitations of body mass index. Am J Clin Nutr. (1988) 47:553. doi: 10.1093/ajcn/47.3.553 3348165

[7] Piche ME Tchernof A Despres JP. Obesity phenotypes, diabetes, and cardiovascular diseases. Circ Res. (2020) 126:1477–500. doi: 10.1161/CIRCRESAHA.120.316101 32437302

[8] Badran M Joseph V. Sleep apnea and diet-induced obesity-the female advantage on the spotlight. Sleep. (2023) 46:zsad174. doi: 10.1093/sleep/zsad174, PMID: 37392414 PMC10424158

[9] Niazi S Moshirfar M Dastjerdi MH Niazi F Doroodgar F Ambrósio R Jr. Association between obesity and age-related cataract: an updated systematic review and dose-response meta-analysis of prospective cohort studies. Front Nutr. (2023) 10:1215212. doi: 10.3389/fnut.2023.1215212 38357464 PMC10866009

[10] Zhang QY Tie LJ Wu SS Lv PL Huang HW Wang WQ. Overweight, obesity, and risk of age-related macular degeneration. Invest Ophthalmol Vis Sci. (2016) 57:1276–83. doi: 10.1167/iovs.15-18637 26990164

[11] Pihlblad MS Schaefer DP. Eyelid laxity, obesity, and obstructive sleep apnea in keratoconus. Cornea. (2013) 32:1232–6. doi: 10.1097/ICO.0b013e318281e755, PMID: 23471083

[12] Mohaghegh S Kangari H Masoumi SJ Bamdad S Rahmani S Abdi S. Prevalence and risk factors of keratoconus (including oxidative stress biomarkers) in a cohort study of shiraz university of medical science employees in Iran. BMC Ophthalmol. (2023) 23:188. doi: 10.1186/s12886-023-02934-0, PMID: 37106365 PMC10142163

[13] Eliasi E Bez M Megreli J Avramovich E Fischer N Barak A. The association between keratoconus and body mass index: a population-based cross-sectional study among half a million adolescents. Am J Ophthalmol. (2021) 224:200–6. doi: 10.1016/j.ajo.2020.11.021, PMID: 33309695

[14] Ren S Tu R Xu L Gu Y Fan Q Wang Q. A high body mass index strengthens the association between the time of eye rubbing and keratoconus in a Chinese population: a case control study. BMC Public Health. (2023) 23:2032. doi: 10.1186/s12889-023-16937-5, PMID: 37853356 PMC10585765

[15] Wang H Zhang Z Zou L Zhang J Jia Z Zhao L. Peripheral artery disease mediating the effect of metabolic syndrome related diseases on lower limb ulcers: Mendelian randomization analysis. Front Endocrinol. (2024) 15:1345605. doi: 10.3389/fendo.2024.1345605, PMID: 38435749 PMC10905962

[16] Yengo L Sidorenko J Kemper KE Zheng Z Wood AR Weedon MN. Meta-analysis of genome-wide association studies for height and body mass index in approximately 700000 individuals of European ancestry. Hum Mol Genet. (2018) 27:3641–9. doi: 10.1093/hmg/ddy271, PMID: 30124842 PMC6488973

[17] Loh PR Kichaev G Gazal S Schoech AP Price AL. Mixed-model association for biobank-scale datasets. Nat Genet. (2018) 50:906–8. doi: 10.1038/s41588-018-0144-6, PMID: 29892013 PMC6309610

[18] Barton AR Sherman MA Mukamel RE Loh PR. Whole-exome imputation within UK Biobank powers rare coding variant association and fine-mapping analyses. Nat Genet. (2021) 53:1260–9. doi: 10.1038/s41588-021-00892-1, PMID: 34226706 PMC8349845

[19] Sakaue S Kanai M Tanigawa Y Karjalainen J Kurki M Koshiba S. A cross-population atlas of genetic associations for 220 human phenotypes. Nat Genet. (2021) 53:1415–24. doi: 10.1038/s41588-021-00931-x, PMID: 34594039 PMC12208603

[20] Locke AE Kahali B. Genetic studies of body mass index yield new insights for obesity biology. Nature. (2015) 518:197–206. doi: 10.1038/nature14177, PMID: 25673413 PMC4382211

[21] Hoffmann TJ Choquet H Yin J Banda Y Kvale MN Glymour M. A large multiethnic genome-wide association study of adult body mass index identifies novel loci. Genetics. (2018) 210:499–515. doi: 10.1534/genetics.118.301479, PMID: 30108127 PMC6216593

[22] Wood AR Tyrrell J Beaumont R Jones SE Tuke MA Ruth KS. Variants in the FTO and CDKAL1 loci have recessive effects on risk of obesity and type 2 diabetes, respectively. Diabetologia. (2016) 59:1214–21. doi: 10.1007/s00125-016-3908-5, PMID: 26961502 PMC4869698

[23] Berndt SI Gustafsson S Mägi R Ganna A Wheeler E Feitosa MF. Genome-wide meta-analysis identifies 11 new loci for anthropometric traits and provides insights into genetic architecture. Nat Genet. (2013) 45:501–12. doi: 10.1038/ng.2606, PMID: 23563607 PMC3973018

[24] Cox AJ West NP Cripps AW. Obesity, inflammation, and the gut microbiota. Lancet Diabetes Endocrinol. (2015) 3:207–15. doi: 10.1016/S2213-8587(14)70134-2 25066177

[25] Gregor MF Hotamisligil GS. Inflammatory mechanisms in obesity. Annu Rev Immunol. (2011) 29:415–45. doi: 10.1146/annurev-immunol-031210-101322 21219177

[26] Rohm TV Meier DT Olefsky JM Donath MY. Inflammation in obesity, diabetes, and related disorders. Immunity. (2022) 55:31–55. doi: 10.1016/j.immuni.2021.12.013, PMID: 35021057 PMC8773457

[27] Krachmer JH Feder RS Belin MW. Keratoconus and related noninflammatory corneal thinning disorders. Surv Ophthalmol. (1984) 28:293–322. doi: 10.1016/0039-6257(84)90094-8, PMID: 6230745

[28] di Martino E Ali M Inglehearn CF. Matrix metalloproteinases in keratoconus—too much of a good thing? Exp Eye Res. (2019) 182:137–43. doi: 10.1016/j.exer.2019.03.016, PMID: 30910610

[29] Balasubramanian SA Mohan S Pye DC Willcox MD. Proteases, proteolysis and inflammatory molecules in the tears of people with keratoconus. Acta Ophthalmol. (2012) 90:e303–9. doi: 10.1111/j.1755-3768.2011.02369.x, PMID: 22413749

[30] Lema I Sobrino T Duran JA Brea D Diez-Feijoo E. Subclinical keratoconus and inflammatory molecules from tears. Br J Ophthalmol. (2009) 93:820–4. doi: 10.1136/bjo.2008.144253, PMID: 19304583

[31] McMonnies CW. Inflammation and keratoconus. Optom Vis Sci. (2015) 92:e35–41. doi: 10.1097/OPX.0000000000000455 25397925

[32] Mazor R Friedmann-Morvinski D Alsaigh T Kleifeld O Kistler EB Rousso-Noori L. Cleavage of the leptin receptor by matrix metalloproteinase-2 promotes leptin resistance and obesity in mice. Sci Transl Med. (2018) 10:eaah6324. doi: 10.1126/scitranslmed.aah6324, PMID: 30135249 PMC9678493

[33] Sawaguchi S Yue BY Sugar J Gilboy JE. Lysosomal enzyme abnormalities in keratoconus. Arch Ophthalmol. (1989) 107:1507–10. doi: 10.1001/archopht.1989.01070020581044, PMID: 2803101

[34] Shetty R Ghosh A Lim RR Subramani M Mihir K Reshma AR. Elevated expression of matrix metalloproteinase-9 and inflammatory cytokines in keratoconus patients is inhibited by cyclosporine a. Invest Ophthalmol Vis Sci. (2015) 56:738–50. doi: 10.1167/iovs.14-14831, PMID: 25648341

[35] Dou S Wang Q Zhang B Wei C Wang H Liu T. Single-cell atlas of keratoconus corneas revealed aberrant transcriptional signatures and implicated mechanical stretch as a trigger for keratoconus pathogenesis. Cell Discov. (2022) 8:66. doi: 10.1038/s41421-022-00397-z, PMID: 35821117 PMC9276680

[36] McMonnies CW Korb DR Blackie CA. The role of heat in rubbing and massage-related corneal deformation. Cont Lens Anterior Eye. (2012) 35:148–54. doi: 10.1016/j.clae.2012.01.001, PMID: 22309634

[37] Balasubramanian SA Pye DC Willcox MD. Effects of eye rubbing on the levels of protease, protease activity and cytokines in tears: relevance in keratoconus. Clin Exp Optom. (2013) 96:214–8. doi: 10.1111/cxo.12038, PMID: 23496656

[38] Gulkas S Aydin FO Turhan SA Toker AE. In vivo corneal confocal microscopy as a non-invasive test to assess obesity induced small fibre nerve damage and inflammation. Eye. (2023) 37:2226–32. doi: 10.1038/s41433-022-02321-x, PMID: 36443498 PMC10366092

