# Supplementary figures and images for: Exploring the causal relationship between body mass index and keratoconus: a Mendelian randomization study
Source: Front Med (Lausanne). 2024 Jul 10;11:1402108. doi: 10.3389/fmed.2024.1402108 (PMC11266172; doi:10.3389/fmed.2024.1402108)

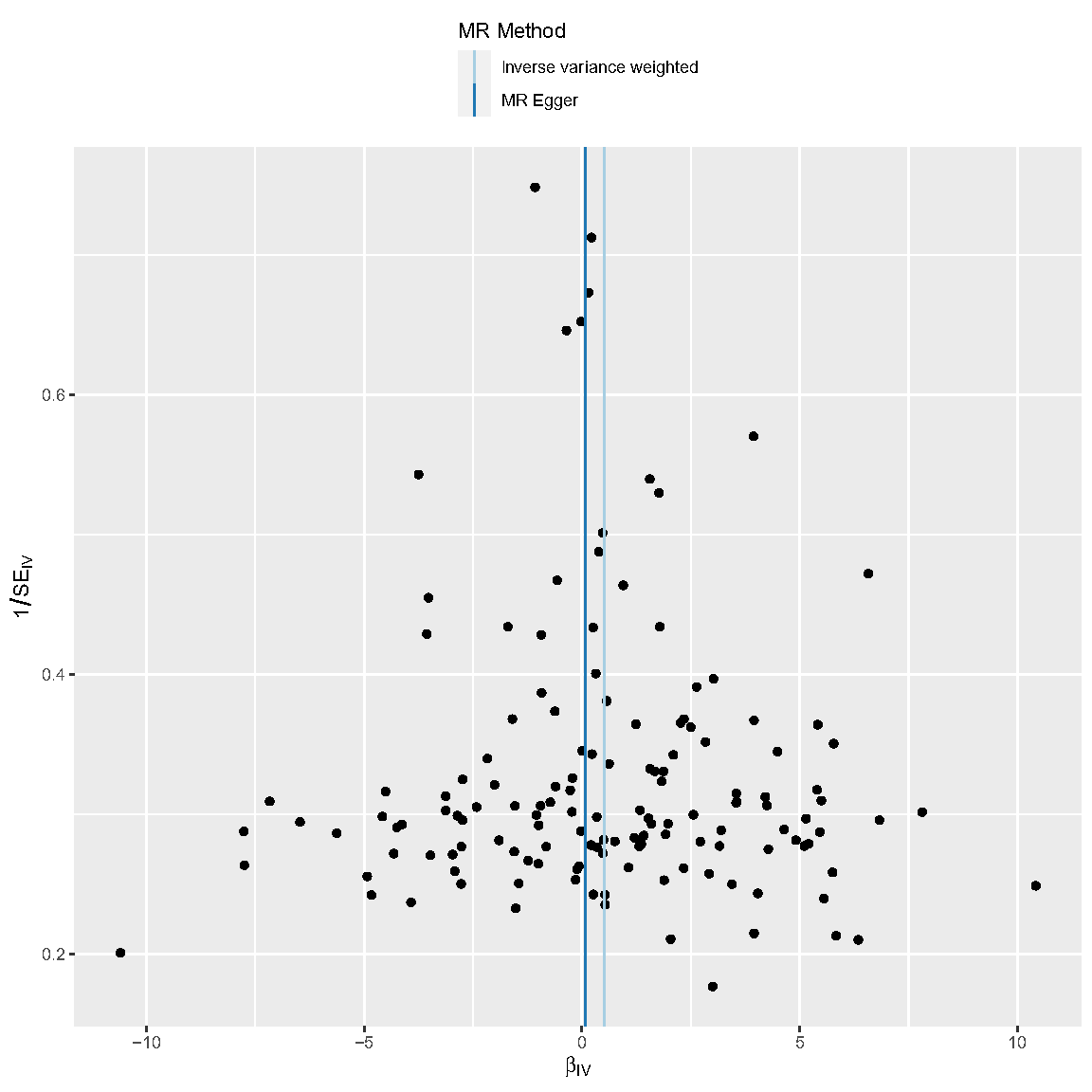

Supplement: Supplementary file 3 [file Image_1.tif]

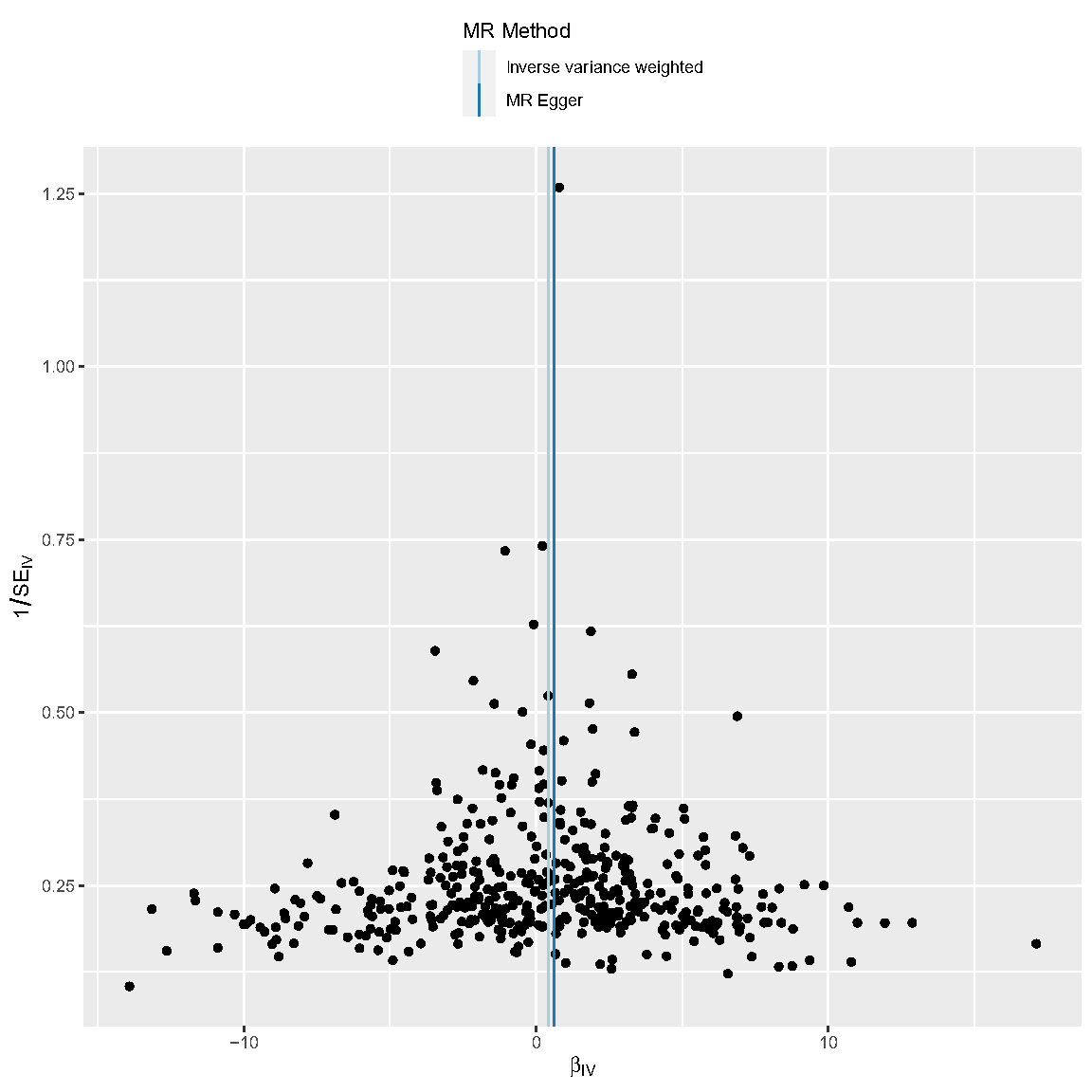

Supplement: Supplementary file 4 [file Image_2.tif]

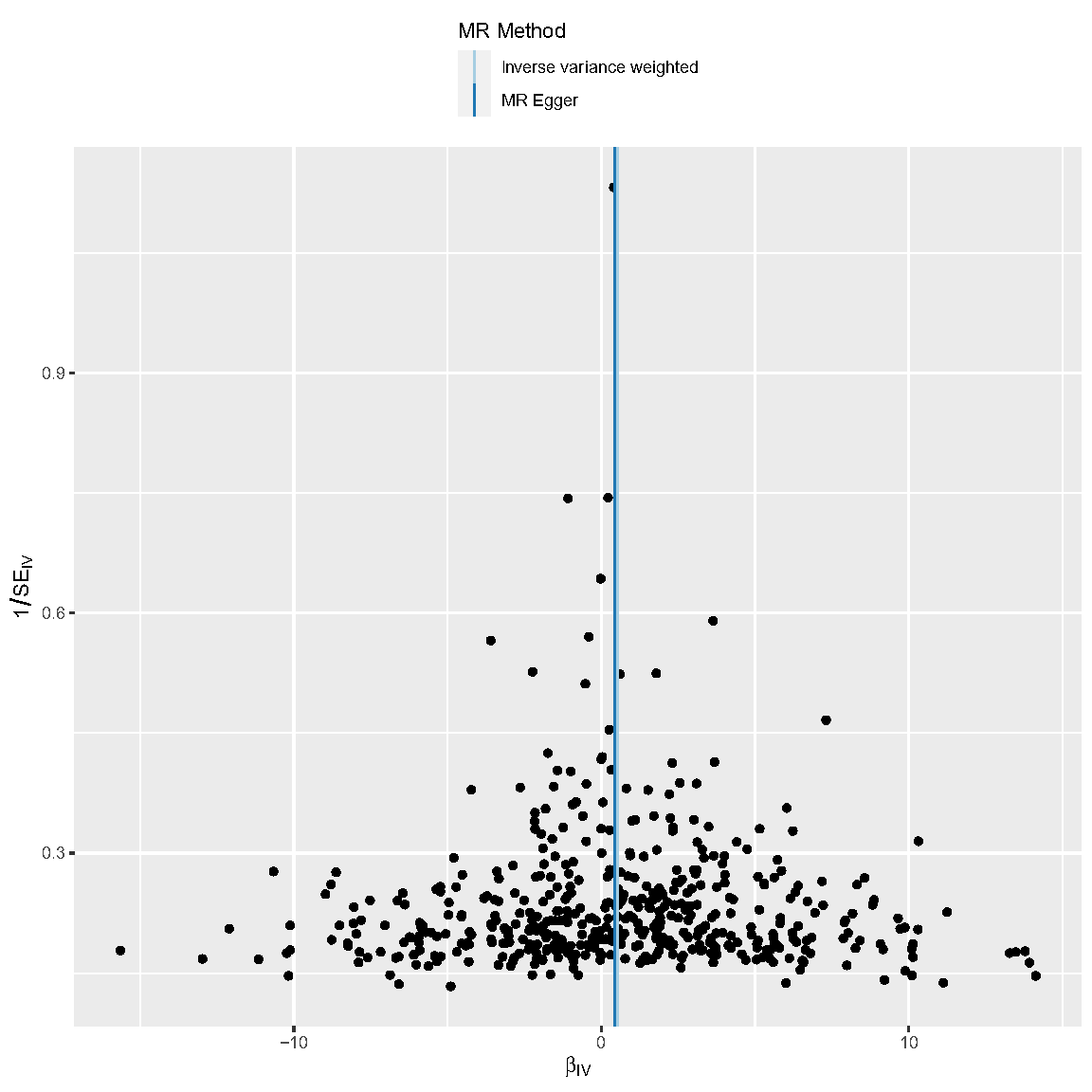

Supplement: Supplementary file 5 [file Image_3.tif]

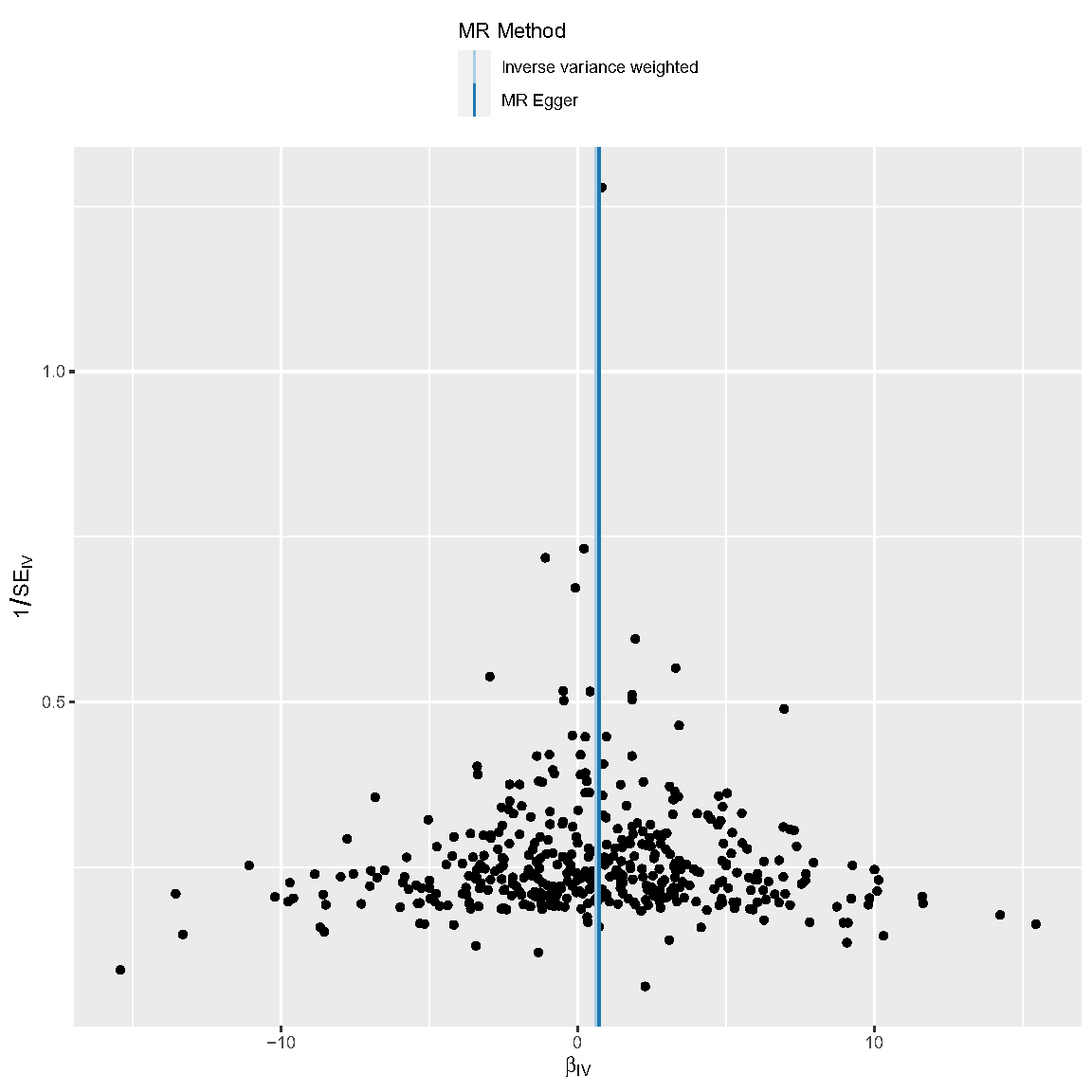

Supplement: Supplementary file 6 [file Image_4.tif]
